# On the limitations of large language models in clinical diagnosis

**DOI:** 10.1101/2023.07.13.23292613

**Published:** 2023-07-14

**Authors:** Justin T Reese, Daniel Danis, J Harry Caulfied, Elena Casiraghi, Giorgio Valentini, Christopher J Mungall, Peter N Robinson

**Affiliations:** 1. Division of Environmental Genomics and Systems Biology, Lawrence Berkeley National Laboratory, Berkeley, CA, 94720, USA.; 2. The Jackson Laboratory for Genomic Medicine, Farmington CT, 06032, USA.; 3. AnacletoLab, Dipartimento di Informatica, Università degli Studi di Milano, Milano, Italy.; 4. ELLIS-European Laboratory for Learning and Intelligent Systems.; 5. Institute for Systems Genomics, University of Connecticut, Farmington, CT 06032, United States.

## Abstract

**Background:**

The potential of large language models (LLM) such as GPT to support complex tasks such as differential diagnosis has been a subject of debate, with some ascribing near sentient abilities to the models and others claiming that LLMs merely perform “autocomplete on steroids”. A recent study reported that the Generative Pretrained Transformer 4 (GPT-4) model performed well in complex differential diagnostic reasoning. The authors assessed the performance of GPT-4 in identifying the correct diagnosis in a series of case records from the New England Journal of Medicine. The authors constructed prompts based on the clinical presentation section of the case reports, and compared the results of GPT-4 to the actual diagnosis. GPT-4 returned the correct diagnosis as a part of its response in 64% of cases, with the correct diagnosis being at rank 1 in 39% of cases. However, such concise but comprehensive narratives of the clinical course are not typically available in electronic health records (EHRs). Further, if they were available, EHR records contain identifying information whose transmission is prohibited by Health Insurance Portability and Accountability Act (HIPAA) regulations.

**Methods:**

To assess the expected performance of GPT on comparable datasets that can be generated by text mining and by design cannot contain identifiable information, we parsed the texts of the case reports and extracted Human Phenotype Ontology (HPO) terms, from which prompts for GPT were constructed that contain largely the same clinical abnormalities but lack the surrounding narrative.

**Results:**

While the performance of GPT-4 on the original narrative-based text was good, with the final diagnosis being included in its differential in 29/75 cases (38.7%; rank 1 in 17.3% of cases; mean rank of 3.4), the performance of GPT-4 on the feature-based approach that includes the major clinical abnormalities without additional narrative texas substantially worse, with GPT-4 including the final diagnosis in its differential in 8/75 cases (10.7%; rank 1 in 4.0% of cases; mean rank of 3.9).

**Interpretation:**

We consider the feature-based queries to be a more appropriate test of the performance of GPT-4 in diagnostic tasks, since it is unlikely that the narrative approach can be used in actual clinical practice. Future research and algorithmic development is needed to determine the optimal approach to leveraging LLMs for clinical diagnosis.

## Introduction

A recent study^1^ reported that the Generative Pretrained Transformer 4 (GPT-4) model performed well in complex differential diagnostic reasoning. They evaluated the performance of GPT-4 on 74 case records from the New England Journal of Medicine (NEJM) published in 2021 and 2022 by sending to GPT-4 a standard prompt, followed by the part of the case report that included the case presentation up to but not including the discussant’s initial response. The authors found that GPT-4 returned the correct diagnosis as a part of its response in 64% of cases, with the correct diagnosis being at rank 1 in 39% of cases.^1^

We examined the influence of linguistic context on the performance of GPT-4 in differential diagnostic reasoning by developing equivalent queries that contained the phenotypic abnormalities described in the original reports without the accompanying narrative text.

## Methods

**Figure 1.**
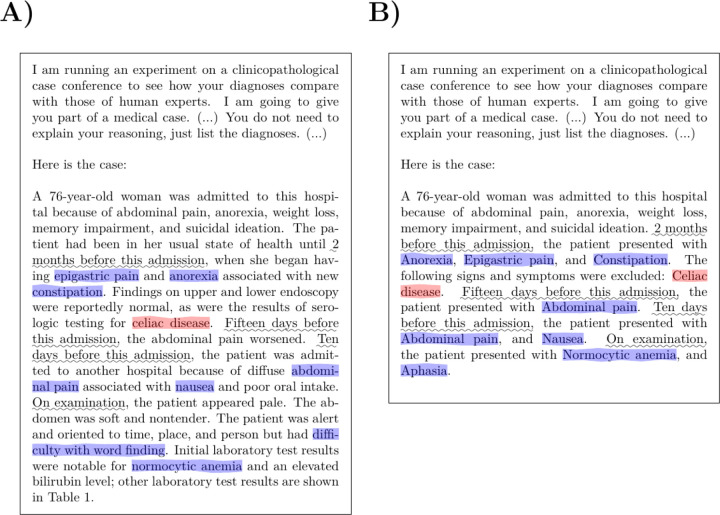
GPT-4 prompt templates. (A) An example showing the narrative template provided to GPT-4 (case 38–2021).^4^ (B) The corresponding example using the simplified, feature-based template. The features in the original text that were mined to generate the feature-based query are highlighted (Observed features in blue and excluded features in red; the phrases introducing the time periods are underlined).. The actual diagnosis in this case was lead poisoning GPT-4 returned the correct diagnosis at rank 11 using the narrative query and did not return any related diagnosis with the feature based query. In this example, the case presentation is relatively short; in many of the analyzed cases, the presentation had a length of a page or longer.

We included NEJM case reports from Case 2–2021 to 40–2022 in our study, omitting 5 of 80 case reports that did not describe a diagnostic dilemma. The text representing the initial clinical presentation was taken from the documents by extracting the first discussant’s section. We appended this text to the standardized prompt as described in the recent study,^1^ and used the OntoGPT^2^ tool to query GPT-4. To assess the influence of the narrative context of the case reports, we extracted the clinical abnormalities as human phenotype ontology (HPO) terms.^3^ Observed and excluded abnormalities were included in the prompt using a standard template, whereby the clinical features were arranged according to the time point of presentation (Figure 1).

## Results

**Figure 2.**
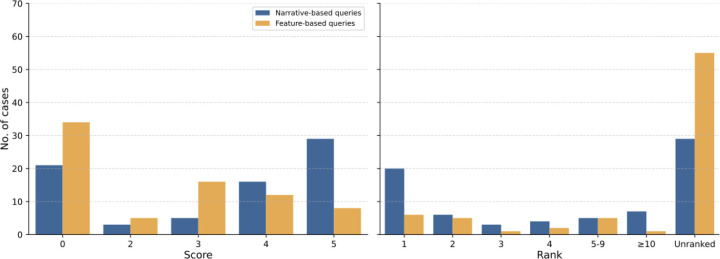
Performance of GPT-4 in diagnostic challenges using narrative and feature-based queries. (A) A histogram of scores (0–5) denoting accuracy of GPT-4 diagnosis using narrative (blue bars) and extracted features (orange bars) from NEJM case reports. (B) A histogram of the ranks of the correct diagnosis in the differential diagnosis produced by GPT-4 in cases where the score was 4 (nearly the correct diagnosis) or 5 (correct diagnosis) using narrative (blue bars) and extracted features (orange bars) from NEJM case reports. The count of unranked cases (scores 0–3) are shown for comparison. The results were assigned scores using the same scale as in the previous study.^1^ Results were scored independently by three coauthors (P.N.R, D.D., J.T.R.) and disagreements were resolved by consensus. 5 = the actual diagnosis was suggested in the differential; 4 = the suggestions included something very close, but not exact; 3 = the suggestions included something closely related that might have been helpful; 2 = the suggestions included something related, but unlikely to be helpful; 0 = no suggestions close to the target diagnosis.

We presented GPT-4 with the original description by the primary discussant (narrative approach), and observed that GPT-4 included the final diagnosis in its differential in 29/75 cases (38.7%; rank 1 in 17.3% of cases; mean rank of 3.4). These results are similar to but not identical to those of the above mentioned study, perhaps because of the stochasticity of the GPT-4 algorithm or changes to the application subsequent to the original study. We then tested the feature-based approach that includes the major clinical abnormalities without additional narrative text. Here, GPT-4 included the final diagnosis in its differential in 8/75 cases (10.7%; rank 1 in 4.0% of cases; mean rank of 3.9) (Figure 2).

## Discussion

The potential of large language models (LLM) such as GPT has been a subject of debate, with some ascribing near sentient abilities to the models and others claiming that LLMs merely perform “autocomplete on steroids”. For the purpose of applying LLMs to the problem of clinical diagnosis, it is important to realize that LLMs generate text based on patterns learned from huge amounts of training texts^5^. LLMs such as GPT-4 do not possess an explicit model of medical domain knowledge and do not perform a symbolic human-like reasoning, but instead performs autocompletion by implicitly learning medical domain knowledge from the data.

We compared the performance of GPT-4 on the original narrative texts and simplified versions of the cases in which only clinical features representable by HPO terms are presented to GPT 4. The performance on the feature-based queries was substantially worse than that of the narrative queries (Figure 2). We consider the feature-based queries to be a more appropriate test of the performance of GPT-4 in diagnostic tasks, since it is unlikely that the narrative approach can be used in actual clinical practice. NEJM-style clinical narratives are not readily available for most cases and EHR texts cannot be transmitted across the internet without violating privacy regulations. In contrast, it is straightforward to generate a feature-based list of clinical problems, symptoms, and other abnormalities that can be used to generate a prompt for GPT. Currently, GPT-4 is not available for installation within medical centers, and it remains an open question as to whether smaller models, eventually embedding structured information, will demonstrate comparable performance. A possible solution could consist in coupling LLMs with a formal representation of medical knowledge, for example using biomedical knowledge graphs.^6^ Future research and algorithmic development is needed to determine the optimal approach to leveraging LLMs for clinical diagnosis.

## Supplementary Material

Supplement 1

Supplement 2
