# Supplementary material for: On the limitations of large language models in clinical diagnosis
Source: medRxiv. 2023 Jul 14:2023.07.13.23292613. Preprint. [Version 1] doi: 10.1101/2023.07.13.23292613 (PMC10370243; doi:10.1101/2023.07.13.23292613)
Supplement: Supplementary file 1 [file media-1.docx]

**Supplement**

Current a random collection of stuff that needs to be put together into a supplement

**Parsing of case reports**

PDF files of 80 New England Journal of Medicine case reports from Case 2-2021 to 40-2022 were downloaded. The texts were extracted with a Python script (See project documentation at [https://github.com/monarch-initiative/phenopacket2prompt](https://github.com/monarch-initiative/phenopacket2prompt)). Five case reports were omitted because they did not describe a diagnostic dilemma (Table S1).

| PMID | Author | Title | Reason |
| --- | --- | --- | --- |
| 34496178 | Neilan | Case 27-2021: A 16-Year-Old Boy Seeking Human Immunodeficiency Virus Prophylaxis | Describes management and not differential diagnosis |
| 33730458 | Goyal | Case 8-2021: A 34-Year-Old Woman with Cholangiocarcinoma | Scant information in case presented by the first discussant. Focus on imaging |
| 33913642 | Koren | Case 13-2021: A Newborn Girl with a Neck Mass | Scant information in case presented by the first discussant. Focus on imaging |
| 34587390 | Lindsay | Case 30-2021: A 47-Year-Old Man with Recurrent Unilateral Head and Neck Pain | Scant information in case presented by the first discussant. Focus on imaging |
| 34670047 | Carpenter | Case 32-2021: A 14-Year-Old Girl with Swelling of the Jaw and Hypercalcemia. | Scant information in case presented by the first discussant. Focus on imaging |

**Table S1**. Case reports omitted from the analysis.

The text that represented the initial clinical presentation was taken from the documents by extracting the first discussant’s section (starting with “Dr. ---:”) up to but not including the response of the second discussant in the Presentation of Case section.

We wrote a Java application to extract clinical features from the texts. The application is freely available under an open source (MIT) license at [https://github.com/monarch-initiative/phenopacket2prompt](https://github.com/monarch-initiative/phenopacket2prompt). 

Prior to using the Java application, we extracted the text from the PDF files using the PyMuPDF library (https://pymupdf.readthedocs.io/en/latest/intro.html).

1. Ingest the text files that were produced by the Python script

2. Strip the text of extraneous symbols introduced by the PDF extraction

3. Extract the text of the first discussant

4. Divide this text up into time periods by using regular expressions to match phrases such as “2 weeks prior to admission”.

5. For each time period, use text mining to identify Human Phenotype Ontology (HPO) terms using the fenominal application ([https://github.com/monarch-initiative/fenominal](https://github.com/monarch-initiative/fenominal))

6. Format the text using a standard pattern.

7. Embed the clinical text within a standard prompt. 

Examples of the narrative prompt and the HPO-based feature prompt are shown in Figures S1 and S2.

| I am running an experiment on a clinicopathological case conference to see how your diagnoses compare with those of human experts. I am going to give you part of a medical case. These have all been published in the New England Journal of Medicine. You are not trying to treat any patients. As you read the case, you will notice that there are expert discussants giving their thoughts. In this case, you are “Dr. GPT-4,” an Al language model who is discussing the case along with human experts. A clinicopathological case conference has several unspoken rules. The first is that there is most often a single definitive diagnosis (though rarely there may be more than one), and it is a diagnosis that is known today to exist in humans. The diagnosis is almost always confirmed by some sort of clinical pathology test or anatomic pathology test, though in rare cases when such a test does not exist for a diagnosis the diagnosis can instead be made using validated clinical criteria or very rarely just confirmed by expert opinion. You will be told at the end of the case description whether a diagnostic test/tests are being ordered, which you can assume will make the diagnosis/diagnoses. After you read the case, I want you to give two pieces of information. The first piece of information is your most likely diagnosis/diagnoses. You need to be as specific as possible -- the goal is to get the correct answer, not a broad category of answers. You do not need to explain your reasoning, just give the diagnosis/diagnoses. The second piece of information is to give a robust differential diagnosis, ranked by their probability so that the most likely diagnosis is at the top, and the least likely is at the bottom. There is no limit to the number of diagnoses on your differential. You can give as many diagnoses as you think are reasonable. You do not need to explain your reasoning, just list the diagnoses. Again, the goal is to be as specific as possible with each of the diagnoses. Do you have any questions, Dr. GPT-4? Here is the case: A 65-year-old man was admitted to this hospital because of bilateral eye pain and decreased vision. The patient had been in his usual state of health until 8 days before this admission, when constant aching pain developed in the left eye and the left side of the head. During the next few days, the vision in the left eye gradually became blurry. Three days before this admission, redness developed in the right eye. The patient used tetrahydrozoline eye drops, but symptoms did not abate. One day before this admission, pain developed in the right eye that was similar to the pain in the left eye. The patient sought evaluation by his primary care physician, who recommended that he present to the emergency department of this hospital for further evaluation. On arrival, the patient rated the eye pain and headache at 2 on a scale of 0 to 10 (with 10 indicating the most severe pain). The pain was not worse with eye movement or accompanied by flashing lights, floaters, or discharge. The patient reported no trauma but noted weight loss of 4.5 kg in the past 2 weeks, as well as pain with chewing food. He did not have scalp tenderness, fever, chills, nausea, vomiting, numbness, weakness, or slurred speech. The patient had a history of Waldenström’s macroglobulinemia, which had been diagnosed 2 years before this admission and treated with plasmapheresis, dexamethasone, and four cycles of bortezomib (with discontinuation of bortezomib after 4 months because of peripheral neuropathy), as well as six cycles of bendamustine. During the 9 months that followed the completion of treatment with bendamustine, there was active surveillance of the blood IgM level. One month before this admission, the IgM level was 1001 mg per deciliter (reference range, 53 to 334), and biopsy of the bone marrow revealed persistent disease. One week before this admission, the patient took his first dose of ibrutinib, which had been prescribed 3 months earlier.Other medical history included latent tuberculosis, depression, anxiety, prediabetes, folate deficiency, and seasonal allergies. Medications included ibrutinib, isoniazid, pyridoxine, sertraline, citalopram, hydroxyzine, cetirizine, fluticasone nasal spray, and a multivitamin. Rituximab had caused anaphylaxis. The patient did not smoke tobacco, use illicit drugs, or drink alcohol. He was originally from Central America and had immigrated to the United States 50 years earlier; 1 month earlier, he had moved from the West Coast to an urban area of New England. He lived in an apartment with his niece and cousins and worked in construction. His family history was unknown. On examination, the temperature was 36.3°C, the blood pressure 104/69 mm Hg, the pulse 60 beats per minute, the respiratory rate 18 breaths per minute, and the oxygen saturation 97% while the patient was breathing ambient air. Visual acuity was 20/20 in the right eye and 20/100 in the left eye with correction. On confrontation testing, visual fields in the left eye were deficient in the inferior temporal and nasal aspects. On the Ishihara color test, all plates were identified with the right eye but only the control plate was identified with the left eye. The pupils were symmetric and reactive to light; an afferent pupillary defect was present in the left eye. There was no proptosis. Subconjunctival hemorrhage was present in the right eye; pterygia were present in both eyes. On funduscopic examination, the optic disk in the left eye was edematous. Results of tests of cranial nerve function, power, sensation, reflexes, coordination, and gait were normal. The blood hemoglobin level was 12.5 g per deciliter (reference range, 13.5 to 17.5), the hematocrit 39.8% (reference range, 41.0 to 53.0), the white-cell count 8090 per microliter (reference range, 4500 to 11,000), and the platelet count 131,000 per microliter (reference range, 150,000 to 400,000). The IgM level was 540 mg per deciliter; the level of IgM kappa M component in the gamma region was 0.36 g per deciliter. Blood levels of electrolytes were normal, as were the results of tests of kidney and liver function. Tests for Lyme disease, syphilis, toxoplasmosis, and bartonellosis were negative. Tests for human immunodeficiency virus (HIV) type 1 p24 antigen and antibodies to HIV types 1 and 2 were negative. Other laboratory test results are shown in Table 1. |
| --- |
| **Figure S1**. Narrative prompt for Case 6-2021: A 65-Year-Old Man with Eye Pain and Decreased Vision. (Bouffard MA, et al., PMID: 33626257). |

| I am running an experiment on a clinicopathological case conference to see how your diagnoses compare with those of human experts. I am going to give you part of a medical case. These have all been published in the New England Journal of Medicine. You are not trying to treat any patients. As you read the case, you will notice that there are expert discussants giving their thoughts. In this case, you are “Dr. GPT-4,” an Al language model who is discussing the case along with human experts. A clinicopathological case conference has several unspoken rules. The first is that there is most often a single definitive diagnosis (though rarely there may be more than one), and it is a diagnosis that is known today to exist in humans. The diagnosis is almost always confirmed by some sort of clinical pathology test or anatomic pathology test, though in rare cases when such a test does not exist for a diagnosis the diagnosis can instead be made using validated clinical criteria or very rarely just confirmed by expert opinion. You will be told at the end of the case description whether a diagnostic test/tests are being ordered, which you can assume will make the diagnosis/diagnoses. After you read the case, I want you to give two pieces of information. The first piece of information is your most likely diagnosis/diagnoses. You need to be as specific as possible -- the goal is to get the correct answer, not a broad category of answers. You do not need to explain your reasoning, just give the diagnosis/diagnoses. The second piece of information is to give a robust differential diagnosis, ranked by their probability so that the most likely diagnosis is at the top, and the least likely is at the bottom. There is no limit to the number of diagnoses on your differential. You can give as many diagnoses as you think are reasonable. You do not need to explain your reasoning, just list the diagnoses. Again, the goal is to be as specific as possible with each of the diagnoses. Do you have any questions, Dr. GPT-4? Here is the case: A 65-year-old man was admitted to this hospital because of bilateral eye pain and decreased vision. 8 days before this admission, the patient presented with Pain. One month before this admission, the patient presented with Depression, Anxiety, Reduced blood folate concentration, Seasonal allergy, Anaphylactic shock, Subconjunctival hemorrhage, Pterygium, and Immunodeficiency. The following signs and symptoms were excluded: Proptosis. One day before this admission, the patient presented with Pain, Pain, Ocular pain, Headache, Pain, Pain, Pain, and Peripheral neuropathy. The following signs and symptoms were excluded: Weight loss, Scalp tenderness, Fever, Chills, Nausea and vomiting, Hypoesthesia, and Slurred speech. Three days before this admission, the patient presented with Ptosis. |
| --- |
| **Figure S2**. Feature-based prompt for Case 6-2021: A 65-Year-Old Man with Eye Pain and Decreased Vision. (Bouffard MA, et al., PMID: 33626257). |

**Script for submitting reports to GPT-4**

def run_analysis(input_data_dir, output_directory, correct_diagnosis_file,

model="gpt-4"):

# Create the output TSV file name

output_file_name = input_data_dir.strip(os.sep).split(os.sep)[-1] + "_results.tsv"

output_file_path = os.path.join(output_directory, output_file_name)

# parse correct diagnosis file

def parse_diagnosis_file(file_path):

result_dict = {}

with open(file_path, 'r') as file:

for line in file:

line = line.strip()

if line:

key, value = line.split('\t')

result_dict[key] = value

return result_dict

correct_diagnosis_dict = parse_diagnosis_file(correct_diagnosis_file)

# Write the header to the output TSV file

with open(output_file_path, "w", encoding="utf-8") as tsv_file:

tsv_file.write("input file name\tcorrect diagnosis\tgpt diagnosis\n")

for filename in os.listdir(input_data_dir):

if filename.endswith(".txt"):

file_path = os.path.join(input_data_dir, filename)

if filename.split('-')[0] in correct_diagnosis_dict:

correct_diagnosis = correct_diagnosis_dict[filename.split('-')[0]]

else:

correct_diagnosis = \

f"Couldn't find {filename} in correct diagnosis file"

with open(file_path, mode='r', encoding="utf-8") as txt_file:

prompt = txt_file.read()

ai = OpenAIClient()

ai.model = model

try:

gpt_diagnosis = ai.complete(prompt)

except openai.error.InvalidRequestError as e:

gpt_diagnosis = "OPENAI API CALL FAILED"

# Write the result to the output TSV file

tsv_file.write(f"{filename}\t{correct_diagnosis}\t\"{gpt_diagnosis}\"\n")
